# The Quality and Reliability of Online Videos as an Information Source of Public Health Education for Stroke Prevention in Mainland China: Electronic Media–Based Cross-Sectional Study

**DOI:** 10.2196/64891

**Published:** 2025-07-21

**Authors:** Rongguang Ge, Haoyi Dai, Chicheng Gong, Yuhong Xia, Rui Wang, Jiaping Xu, Shoujiang You, Yongjun Cao

**Affiliations:** 1 Department of Neurology and Clinical Research Center of Neurological Disease, The Second Affiliated Hospital of Soochow University, 1055 Sanxiang Road, Suzhou, China, 86 0512-68282030

**Keywords:** credibility, quality, online video, stroke, prevention, public health education

## Abstract

**Background:**

Stroke has become a leading cause of death and disability worldwide, resulting in a significant loss of healthy life years and imposing a considerable economic burden on patients, their families, and caregivers. However, despite the growing role of online videos as an emerging source of health information, the credibility and quality of stroke prevention education videos, especially those in Chinese, remain unclear.

**Objective:**

This study aims to assess the basic characteristics, overall quality, and reliability of Chinese-language online videos related to public health education on stroke prevention.

**Methods:**

We systematically searched and screened stroke prevention–related video resources from 4 popular Chinese domestic video platforms (Bilibili, Douyin, Haokan, and Xigua). General information, including upload date, duration, views, likes, comments, and shares, was extracted and recorded. Two validated evaluation tools were used: the modified DISCERN questionnaire to assess content reliability and the Global Quality Scale (GQS) to evaluate overall quality. Finally, Spearman correlation analysis was conducted to examine potential associations between general video metrics and their quality and reliability.

**Results:**

After searching and screening, a total of 313 eligible videos were included for analysis: 68 from Bilibili, 74 from Douyin, 86 from Haokan, and 85 from Xigua. Among these, 113 (36.1%) were created by health care professionals, followed by news agencies (n=95, 30.4%) and general individual users (n=40, 12.8%). The median scores for the modified DISCERN and GQS were 2 and 3, respectively, suggesting that the included stroke prevention–related videos were relatively unreliable and of moderate quality. Most videos focused on primary stroke prevention and commonly recommended adopting a healthy diet; engaging in physical activity; and managing blood pressure, glucose, and lipid levels. Additionally, videos with longer durations and more comments tended to be more reliable and of higher quality. A positive association was also observed between video quality and reliability.

**Conclusions:**

Overall, the quality and reliability of Chinese-language online videos as a source of stroke prevention information remain unsatisfactory and should be approached with caution by viewers. To address this issue, several measures should be implemented, including establishing an online monitoring and correction system, strengthening the video review process through collaboration with health care professionals, and encouraging more selective and cautious sharing of controversial content. These steps are essential to help curb the spread of online misinformation and minimize its ongoing impact.

## Introduction

Stroke, which is primarily classified into ischemic and hemorrhagic types, is an extremely lethal and disabling neurological disease, accounting for approximately 7.3 million deaths and 160.5 million years of healthy life lost worldwide in 2021 alone [1]. China, as one of the largest developing countries, faces an increasingly severe challenge from the burden of stroke due to its large population and rapid aging [2].

In the pursuit of better combating this growing threat, the primary prevention of first stroke and secondary prevention of recurrent stroke have emerged as critical strategies and are now recognized as essential components of global stroke control [3,4]. As a result, prevention-oriented public and patient education is increasingly emphasized, with various specific educational methods, such as standardized courses [5], peer education [6], and community-based programs [7], proven to significantly improve stroke management quality and help mitigate rising medical expenditures.

Over the past few decades, the continuous development of the internet and the growing ownership of mobile devices have inevitably created new opportunities for education on cerebrovascular disease. In particular, online video-sharing platforms, known for their easy accessibility and user-friendly interfaces, have significantly transformed how health information is disseminated and accessed by the public [8,9]. Compared with traditional educational methods involving 1-on-1 teaching and print materials, online video-based disease education may offer a distinct advantage by presenting graphical content that is easier for audiences to absorb and retain. For example, YouTube (Google LLC/Alphabet Inc) has been found to provide useful video resources that compile information about stroke treatment options for patients and their families [10].

However, despite these benefits, the drawbacks of online video-based patient education cannot be ignored. Videos created by amateurs may contain numerous rumors, hoaxes, and misinformation that lack a solid scientific basis [11,12]. Consequently, it remains an arduous and complicated task for most patients with stroke and general users to fully evaluate the overall quality and reliability of stroke-related videos automatically recommended by search engines [13]. Given that Google (Alphabet Inc) has withdrawn its operations from Mainland China, YouTube has been unavailable to the majority of the Chinese population since 2010. Nevertheless, some Chinese domestic video-sharing platforms, such as TikTok (ByteDance Ltd), have gained tremendous popularity across the nation with their engaging content and user interaction.

Although several previous studies have investigated the quality of stroke-related materials on YouTube, TikTok, and similar platforms, showing variable reliability and adherence to evidence-based information, most of this literature originates from English-speaking contexts [10,14-17]. For instance, a systematic review by Garg et al [18] assessed social media’s role in stroke education globally, while other studies have evaluated video content acquired in countries such as India [14] and Turkey [17]. However, research focusing on Chinese-language video content remains limited, despite the heavy burden of stroke and the widespread use of social media in China.

Therefore, our research team aimed to systematically assess the overall quality and reliability of online videos related to stroke prevention in Mainland China by searching for and extracting relevant Chinese-language videos from the 4 most popular online video platforms. This study seeks to address the existing academic gap and help future viewers more appropriately select stroke-related video resources.

## Methods

### Search Strategy

In this cross-sectional study based on electronic media, the following search terms were used: “中风” “脑卒中” (Chinese for “stroke”), “脑梗死” (Chinese for “ischemic stroke”), “脑出血” (Chinese for “hemorrhagic stroke”), and “预防” (Chinese for “prevention”). These terms were used to retrieve the top 100 videos automatically recommended by the default overall ranking on 4 popular Chinese-language online video-sharing platforms: Bilibili (Bilibili Inc/Shanghai Hode Information Technology Co, Ltd/Sony Group Corporation) [19], Douyin (ByteDance Ltd) [20] (the Chinese counterpart of TikTok), Haokan Video (Baidu, Inc) [21], and Xigua Video (ByteDance Ltd) [22]. A brief introduction to these 4 Chinese-language video platforms is provided in Table 1.

**Table 1.** A brief introduction to Bilibili, Douyin, Haokan Video, and Xigua Video.

Online video platform	History and introduction
Bilibili	Founded by Shanghai Kuanyu Digital Technology on June 26, 2009, Bilibili has become a cultural community and video platform known for its bullet comment feature and strong popularity among China’s younger generation.
Douyin (Chinese counterpart of TikTok)	Founded by ByteDance in 2016, Douyin (the Chinese counterpart of TikTok) is a short video platform aimed at young audiences in China. It encourages users to record and share their daily lives using smartphones or other available filming equipment.
Haokan Video	Founded by Baidu in December 2017, Haokan Video is currently positioned as the “Comprehensive Short Video Flagship” among Baidu’s products. The platform is committed to developing a broad short video ecosystem that spans knowledge, lifestyle, health, culture, history, science popularization, technology, emotions, news, film, television, and other domains.
Xigua Video	Founded by ByteDance in 2017, Xigua Video has become a leading domestic professional + user-generated content platform in China. It delivers high-quality content to diverse audiences through personalized recommendations, while promoting creative diversity and making it easy for users to share their videos.

The selection of search keywords was based on both medical terminology and commonly used lay terms for stroke within the Chinese context to ensure relevance and comprehensiveness. This approach aimed to capture a broad spectrum of videos that users might realistically encounter. For example, we included both formal terms such as “脑卒中” (formal medical terminology for “stroke” in the Chinese language) and more colloquial expressions such as “中风” (colloquial description of “stroke” in the Chinese language), which are widely used in public discourse and online media. In addition, we referred to trending tags and frequently searched phrases related to stroke on Chinese video-sharing platforms to enhance the validity of the search keyword set.

With regard to the automatic recommendation and ranking algorithm, consultation with the customer service agents from the selected platforms indicated that the algorithms are primarily driven by video relevance, followed by a comprehensive evaluation based on popularity metrics such as views and likes. However, more detailed information about the algorithm’s formulas and methodologies is unavailable due to concerns related to commercial confidentiality.

Video retrieval was conducted on a Windows 10 (Microsoft Corporation) personal computer using a newly installed Mozilla Firefox browser (version 121.0.1; Mozilla Foundation and Mozilla Corporation) in Suzhou, Jiangsu, China, within a single day (December 28, 2023) to minimize potential bias from newly uploaded content. To reduce the influence of personalized recommendation algorithms, all cookies, browsing history, and temporary internet files were cleared, and platform accounts were logged out before and between search queries, which were performed in incognito mode.

### Video Selection Criteria

After conducting the searches, the top 100 videos automatically recommended based on the default overall ranking on each video platform were selected and prepared for initial screening. This decision was informed by previous literature indicating that most amateur health seekers rarely browse beyond the first 2 pages of search results [23]. Additionally, most Chinese-language video-sharing platforms limit the number of videos displayed per page to no more than 30. Specifically, Bilibili displays 30 videos per page, Haokan Video 10, Xigua Video 20, and Douyin 20. Therefore, we believe that our sample size (n=100) exceeds the number of videos typically displayed on the first 2 pages (Bilibili, n=60; Haokan Video, n=20; Xigua Video, n=40; and Douyin, n=40), which audiences are most likely to engage with, thereby ensuring representativeness. Furthermore, a sample size of 100 has been widely adopted in similar previous studies [24-26], further supporting the rationale for selecting the top 100 automatically recommended videos for subsequent filtering and analysis.

**Figure 1.**
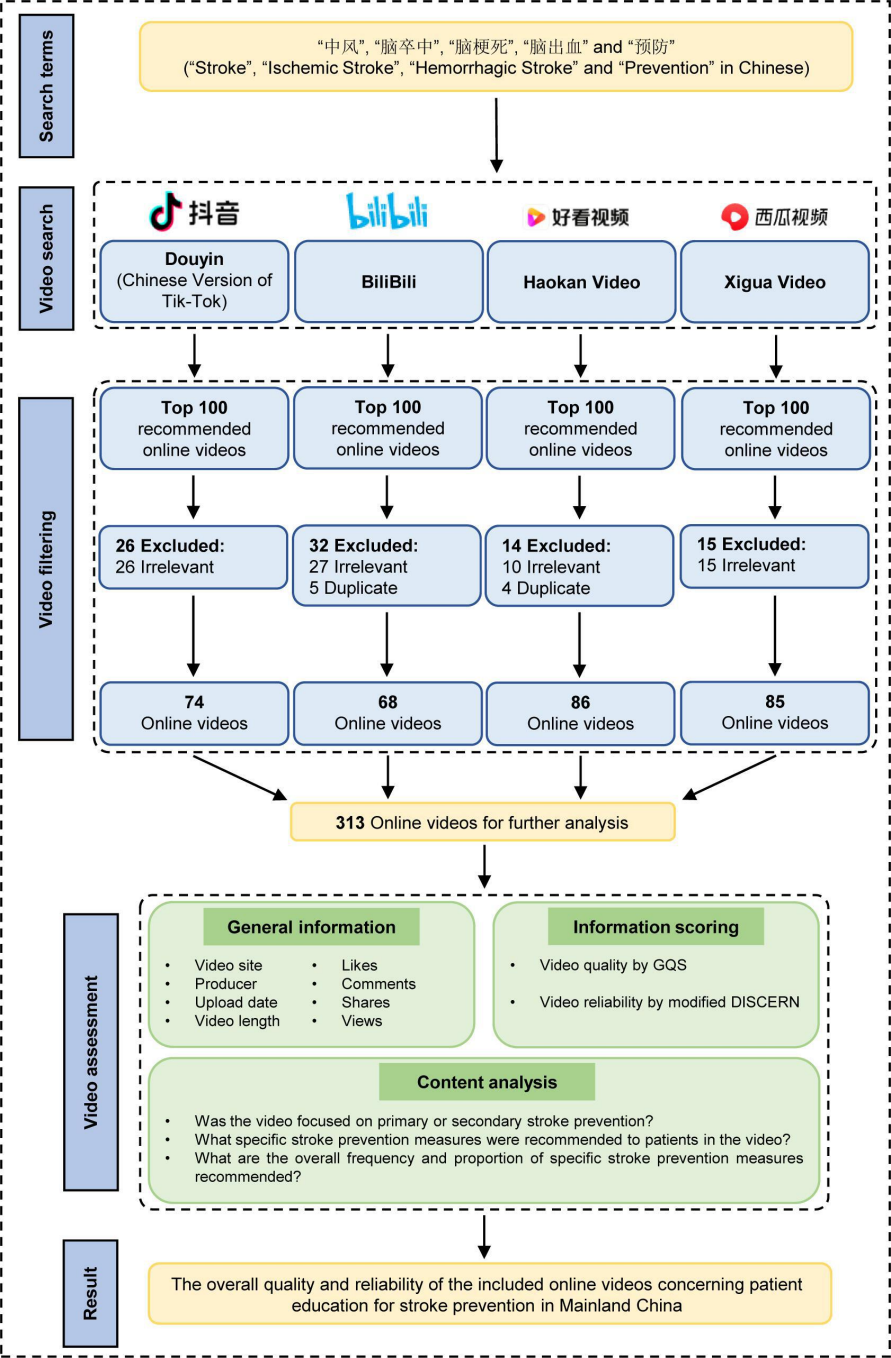
Flowchart of the retrieval and screening process for Chinese-language online videos related to stroke prevention. GQS: Global Quality Scale.

The exclusion criteria were defined as follows: (1) videos not directly related to stroke prevention; (2) duplicated videos repeatedly uploaded to the same platform; (3) videos not in Chinese (including Mandarin, Cantonese, and other dialects); and (4) commercial advertisements. In this study, 78 off-topic videos and 9 duplicate videos uploaded to the same platform were identified and excluded, resulting in a final sample of 313 eligible videos for further analysis (Figure 1). All eligible videos were produced in Mandarin Chinese, and no highly similar or fully duplicate videos were observed across different platforms.

### General Information Extraction

For the included videos, general information, including URLs; authorship; number of views, likes, shares, and comments; upload date; and video length (in seconds), was collected and recorded in an Excel spreadsheet (Microsoft Corporation) by an independent researcher (RGG).

### Classification of Videos

The included videos were categorized based on the identity of their producers as follows: (1) general users (eg, amateurs); (2) health professionals (eg, medical practitioners); (3) science communicators (eg, citizen science storytellers); (4) news agencies (eg, CCTV [China Central Television]); (5) medical organizations (eg, hospital); (6) for-profit organizations (eg, commercial health care–related enterprise); and (7) nonprofit organizations (eg, voluntary patient communities).

For videos classified under health professionals, a further subcategorization was conducted as follows: (1) doctors specializing in neurology within modern evidence-based medicine; (2) doctors specializing in other areas of modern evidence-based medicine; (3) doctors of traditional Chinese medicine (TCM); and (4) other health-related professionals, further specified according to their specialty. Detailed definitions of the authorship categories are provided in Table S1 in Multimedia Appendix 1.

### Video Evaluation Framework

#### Quality and Reliability Assessment

After completing the video retrieval, exclusion, and classification, 2 separate questionnaires were used to quantitatively assess the overall quality and reliability of the included online videos on stroke prevention.

The DISCERN instrument, first introduced in 1999 to evaluate the quality of online information on treatment choices [27], has since been widely validated and applied to assess health-related content on video-sharing platforms [28,29]. In 2012, Singh et al [30] developed a modified version of DISCERN, streamlining the original 16-question format into a 5-question version that focuses on clarity, reliability, bias/balance, provision of additional information sources, and acknowledgment of areas of uncertainty. This modified version has also been extensively tested and is commonly used in previous studies [31-35]. Therefore, in this study, we used the modified DISCERN instrument to assess the reliability of video content based on its 5 items (Table 2). The total score of the modified DISCERN tool ranges from 0 to 5, with higher scores indicating greater reliability (from unreliable to reliable). Furthermore, the GQS, a commonly used 5-point scale ranging from 1 (poor quality) to 5 (excellent quality) for evaluating health-related content on the internet [25,36,37], was also applied to assess the overall quality of the included videos (Table 3).

**Table 2.** Modified DISCERN questionnaire (1 point for every yes and 0 points for no) items and their descriptions.

Item	Description
1	Are the aims clear and achieved?
2	Are reliable sources of information used?
3	Is the information presented balanced and unbiased?
4	Are additional sources of information listed for patient reference?
5	Are areas of uncertainty mentioned?

**Table 3.** Global Quality Scale grades and their descriptions.

Grade	Description
1	Poor quality, poor flow, most information missing; not helpful for patients.
2	Generally poor, some information given; limited use for patients.
3	Moderate quality, some information is adequately discussed, but important topics are missing; somewhat useful to patients.
4	Good quality and flow, most relevant information is covered; useful for patients.
5	Excellent quality and flow; very useful for patients.

Two qualified neurologists (HYD and YHX), both with sufficient medical education and clinical experience, were assigned to independently assess the included online videos using the modified DISCERN and GQS tools. Before formal scoring, both raters thoroughly reviewed and familiarized themselves with the questionnaires to minimize potential biases caused by any misunderstanding of the scoring instruments.

Moreover, the Cohen κ test was used to assess interrater agreement. A value greater than 0.8 was considered indicative of excellent consistency; a value between 0.8 and 0.6 represented substantial consistency; a value between 0.6 and 0.4 indicated moderate consistency; and a value below 0.4 was considered poor consistency [38]. Disagreements between the 2 reviewers were resolved through discussion; if a consensus could not be reached, a senior researcher (YJC) was appointed to make the final decision.

#### Video Content Evaluation

With regard to the video content related to stroke prevention, 3 key aspects were considered, as outlined below: (1) Which stage of stroke prevention was mentioned (primary, secondary, or both)? (2) What specific preventive measures were recommended (including both pharmacological and nonpharmacological approaches)? (3) What were the frequency and proportion of specific stroke prevention measures recommended in the videos?

As for the rationale behind using this 3-item combined evaluation strategy: Identifying the stage of prevention helps determine whether a video emphasizes preventing the initial onset of stroke or preventing recurrence, an area often overlooked in public education. Examining the specific preventive strategies provides insight into whether the content offers accurate and actionable guidance aligned with current clinical guidelines. Lastly, quantifying the frequency and distribution of these strategies allows for an assessment of the consistency and emphasis of public health messaging across different videos and platforms. Together, these 3 items offer a structured lens through which to assess both the breadth and clinical relevance of video content, aligning with the overall goal of evaluating the educational quality and reliability of Chinese-language videos on stroke prevention.

This procedure was performed by another independent reviewer (CCG), and any uncertainties were resolved through discussion with the research team.

### Statistical Analysis

In this study, the Shapiro-Wilk test was used to assess the normality of the quantitative data extracted from the included videos. For nonnormally distributed data, the median and IQR were used for descriptive analysis, whereas for normally distributed data, the mean and SD were reported. Categorical data were presented as absolute numbers and percentages.

Comparisons between 2 groups were performed using the nonparametric Mann-Whitney *U* test, while comparisons among 3 or more groups were conducted using the Kruskal-Wallis *H* test. The Spearman correlation coefficient was then used to examine associations between video variables and quality and reliability scores. For interpretation, a correlation coefficient less than 0.10 was considered negligible; between 0.10 and 0.39, weak; between 0.40 and 0.69, moderate; between 0.70 and 0.89, strong; and greater than 0.90, very strong [39]. Finally, a linear regression model was applied to explore potential temporal trends in the quality and reliability of stroke prevention–related videos over the upload period. A *P* value of <.05 was considered statistically significant. All analyses were performed with the GraphPad Prism software (version 9.5.1; GraphPad Software, Inc).

### Ethical Considerations

This study did not involve human participants or experimental animals; therefore, ethics approval and consent to participate were not applicable. Our study involved the analysis of publicly available data or the use of secondary data that do not contain any personal identifiers, thus ensuring the anonymity and confidentiality of the individuals concerned. Compliance with ethical standards was maintained throughout the research process.

## Results

### Overview

As shown in Figure 1, after the search and screening process, a total of 313 eligible online videos on stroke prevention education were included for further analysis. Of these, 68 were from Bilibili, 74 from Douyin (the Chinese counterpart of TikTok), 86 from Haokan Video, and 85 from Xigua Video.

Overall, as shown in Table 4, the median values among the included videos were as follows: video length, 137 seconds; views, 779; likes, 18; shares, 67; and comments, 1. Regarding the year of video upload, the number of uploaded videos has steadily increased over time, with the majority uploaded in 2023, accounting for 106 out of 313 (33.9%) total videos uploaded. When analyzed by video authorship, health professionals, including doctors specializing in neurology and other fields of modern medicine, doctors of TCM, and pharmacists, collectively created and uploaded 113 stroke prevention–related videos (n=113, 36.1%), followed by news agencies (n=95, 30.4%) and general users (n=40, 12.8%). More detailed descriptive results by video platforms are presented in Table 4.

**Table 4.** The general information, producer identity, quality and reliability scores, content, and recommended stroke prevention strategy of online videos uploaded on Bilibili, Douyin, Haokan Video, and Xigua Video.

Video-sharing platforms	Total	Bilibili	Douyin	Haokan	Xigua
General information					
Number, n	313	68	74	86	85
Length (seconds), median (IQR)	137 (81-213.5)	156 (89-279)	88 (55-138.3)	151.5 (94-213.5)	142 (100-253.5)
Views (n), median (IQR)	779 (336-4565)	352.5 (123-1103)	N/A^a^	1299 (577.3-6996)	922 (355-4369)
Likes (n), median (IQR)	18 (2.5-306.5)	3.5 (1-22.25)	662 (281-4477)	10 (3-64.5)	6 (1-44.5)
Shares (n), median (IQR)	67 (5-327.8)	8 (1-38)	172.5 (69.25-906.8)	N/A^b^	N/A^c^
Comments (n), median (IQR)	1 (0-10)	0 (0-2)	24.5 (7.75-106.3)	0 (0-2)	0 (0-2)
Upload year, n (%)					
2023	106 (33.9)	35 (51.5)	38 (51.4)	21 (24.4)	12 (14.1)
2022	68 (21.7)	15 (22.1)	17 (23.0)	17 (19.8)	19 (22.4)
2021	64 (20.4)	11 (16.2)	13 (17.6)	18 (20.9)	22 (25.9)
2020	43 (13.7)	5 (7.4)	5 (6.8)	17 (19.8)	16 (18.8)
2019	23 (7.3)	2 (2.9)	1 (1.4)	12 (14.0)	8 (9.4)
2018	6 (1.9)	0 (0.0)	0 (0.0)	1 (1.2)	5 (5.9)
2017	3 (1.0)	0 (0.0)	0 (0.0)	0 (0.0)	3 (3.5)
Authorship, n (%)					
General users	40 (12.8)	16 (23.5)	0 (0.0)	12 (14.0)	12 (14.1)
Science communicators	11 (3.5)	10 (14.7)	1 (1.4)	0 (0.0)	0 (0.0)
Health professionals	113 (36.1)	14 (20.6)	60 (81.1)	12 (14.0)	27 (31.8)
Doctors specializing in neurology of modern medicine	32 (10.2)	3 (4.4)	23 (31.1)	0 (0.0)	6 (7.1)
Doctors specializing in other areas of modern medicine	59 (18.8)	9 (13.2)	33 (44.6)	5 (5.8)	12 (14.1)
Doctors of traditional Chinese medicine	19 (6.1)	2 (2.9)	4 (5.4)	7 (8.1)	6 (7.1)
Pharmacists	3 (1.0)	0 (0.0)	0 (0.0)	0 (0.0)	3 (3.5)
Medical organizations	18 (5.8)	7 (10.3)	9 (12.2)	0 (0.0)	2 (2.4)
For-profit organizations	18 (5.8)	6 (8.8)	0 (0.0)	3 (3.5)	9 (10.6)
Nonprofit organizations	18 (5.8)	11 (16.2)	2 (2.7)	1 (1.2)	4 (4.7)
News agencies	95 (30.4)	4 (5.9)	2 (2.7)	58 (67.4)	31 (36.5)
Quality and reliability					
Global Quality Scale score, mean (SD)	3.12 (0.94)	3.41 (0.95)	2.93 (0.83)	2.92 (1.00)	3.26 (0.89)
Global Quality Scale score, median (IQR)	3 (3-4)	3 (3-4)	3 (3-3)	3 (2-3)	3 (3-4)
DISCERN score, mean (SD)	2.47 (0.92)	2.60 (0.87)	2.70 (0.95)	2.11 (0.85)	2.52 (0.91)
DISCERN score, median (IQR)	2 (2-3)	2.5 (2-3)	3 (2-3)	2 (2-3)	2 (2-3)
Content analysis					
Primary stroke prevention, n (%)					
Physical activity	144 (46.0)	36 (52.9)	32 (43.2)	38 (44.2)	38 (44.7)
Healthy diet	145 (46.3)	39 (57.4)	28 (37.8)	38 (44.2)	40 (47.1)
Control blood pressure	158 (50.5)	31 (45.6)	38 (51.4)	40 (46.5)	49 (57.6)
Control blood glucose	127 (40.6)	27 (39.7)	29 (39.2)	34 (39.5)	37 (43.5)
Control blood lipid	129 (41.2)	29 (42.6)	32 (43.2)	34 (39.5)	34 (40.0)
Reduce weight	73 (23.3)	14 (20.6)	21 (28.4)	17 (19.8)	21 (24.7)
Control blood homocysteine	21 (6.7)	6 (8.8)	4 (5.4)	5 (5.8)	6 (7.1)
Quit smoking	120 (38.3)	34 (50.0)	28 (37.8)	23 (26.7)	35 (41.2)
Quit drinking	99 (31.6)	31 (45.6)	15 (20.3)	22 (25.6)	31 (36.5)
Treat atrial fibrillation	33 (10.5)	8 (11.8)	10 (13.5)	6 (7.0)	9 (10.6)
Treat other heart diseases	34 (10.9)	9 (13.2)	13 (17.6)	6 (7.0)	6 (7.1)
Monitor carotid artery stenosis	19 (6.1)	1 (1.5)	11 (14.9)	1 (1.2)	6 (7.1)
Treat migraine	2 (0.6)	1 (1.5)	1 (1.4)	0 (0.0)	0 (0.0)
Treat obstructive sleep apnea-hypopnea syndrome	4 (1.3)	0 (0.0)	3 (4.1)	0 (0.0)	1 (1.2)
Control hypercoagulable state	5 (1.6)	2 (2.9)	1 (1.4)	0 (0.0)	2 (2.4)
Control inflammation or infection	5 (1.6)	0 (0.0)	3 (4.1)	0 (0.0)	2 (2.4)
Oral antiplatelet drugs	22 (7.0)	4 (5.9)	7 (9.5)	5 (5.8)	6 (7.1)
Traditional Chinese herbs	17 (5.4)	2 (2.9)	2 (2.7)	10 (11.6)	3 (3.5)
Acupuncture and moxibustion	1 (0.3)	0 (0.0)	0 (0.0)	0 (0.0)	1 (1.2)
Massage	1 (0.3)	0 (0.0)	0 (0.0)	1 (1.2)	0 (0.0)
Avoid sudden changes in temperature	28 (8.9)	12 (17.6)	2 (2.7)	3 (3.5)	11 (12.9)
Remain emotionally stable	68 (21.7)	20 (29.4)	14 (18.9)	14 (16.3)	20 (23.5)
Secondary stroke prevention, n (%)					
Persistent clinical follow-up	2 (0.6)	0 (0.0)	2 (2.7)	0 (0.0)	0 (0.0)
Healthy diet	13 (4.2)	6 (8.8)	2 (2.7)	3 (3.5)	2 (2.4)
Physical activity	14 (4.5)	6 (8.8)	4 (5.4)	3 (3.5)	1 (1.2)
Quit smoking	13 (4.2)	6 (8.8)	4 (5.4)	2 (2.3)	1 (1.2)
Quit drinking	14 (4.5)	6 (8.8)	5 (6.8)	2 (2.3)	1 (1.2)
Control blood pressure	24 (7.7)	8 (11.8)	7 (9.5)	6 (7.0)	3 (3.5)
Control blood glucose	21 (6.7)	7 (10.3)	7 (9.5)	6 (7.0)	1 (1.2)
Control blood lipid	21 (6.7)	6 (8.8)	7 (9.5)	5 (5.8)	3 (3.5)
Reduce weight	7 (2.2)	2 (2.9)	4 (5.4)	1 (1.2)	0 (0.0)
Control blood homocysteine	1 (0.3)	1 (1.5)	0 (0.0)	0 (0.0)	0 (0.0)
Oral antiplatelet drugs	21 (6.7)	6 (8.8)	6 (8.1)	4 (4.7)	5 (5.9)
Treat atrial fibrillation	7 (2.2)	5 (7.4)	1 (1.4)	1 (1.2)	0 (0.0)
Treat other heart diseases	6 (1.9)	3 (4.4)	2 (2.7)	1 (1.2)	0 (0.0)
Monitor carotid artery stenosis	1 (0.3)	0 (0.0)	1 (1.4)	0 (0.0)	0 (0.0)
Carotid artery stenting	5 (1.6)	3 (4.4)	2 (2.7)	0 (0.0)	0 (0.0)
Avoid sudden change in temperature	1 (0.3)	1 (1.5)	0 (0.0)	0 (0.0)	0 (0.0)
Remain emotionally stable	5 (1.6)	1 (1.5)	1 (1.4)	2 (2.3)	1 (1.2)

a The number of views was not available for the Douyin platform.

b The number of shares was not available for the Haokan platform.

c The number of shares was not available for the Xigua platform.

With regard to the overall quality and reliability of the included online videos, the kappa coefficients for the modified DISCERN and GQS scores, as assessed by 2 independent raters, were 0.71 and 0.73, respectively, indicating substantial interrater agreement. The observed discrepancies were primarily due to 1 rater slightly overestimating the quality and reliability of certain stroke prevention–related videos. However, most of these minor disagreements were resolved through discussion between the 2 raters. In cases where disagreement persisted, a senior researcher was assigned to make the final decision. Taken together, with the median scores for the modified DISCERN and GQS being 2 and 3, respectively, our results suggest that the included videos exhibited relatively poor reliability but moderate quality.

### Video Upload Timeline

The distribution of video uploads over time revealed a clear upward trend. As shown in Table 4, the number of stroke prevention–related videos increased steadily each year. Only a small proportion of videos were uploaded before 2020, with a marked rise beginning in 2021. Notably, the highest number of videos (n=106, 33.9%) was uploaded in 2023 alone, suggesting growing public and institutional engagement with stroke-related health education on video platforms in recent years. This increase may reflect heightened awareness of cerebrovascular disease prevention, evolving content production practices, or rising user engagement with online platforms across China. These temporal patterns underscore the relevance and urgency of evaluating video-based health information, as both the volume and impact of such content continue to grow.

### Quality and Reliability by Video Platform

**Figure 2.**
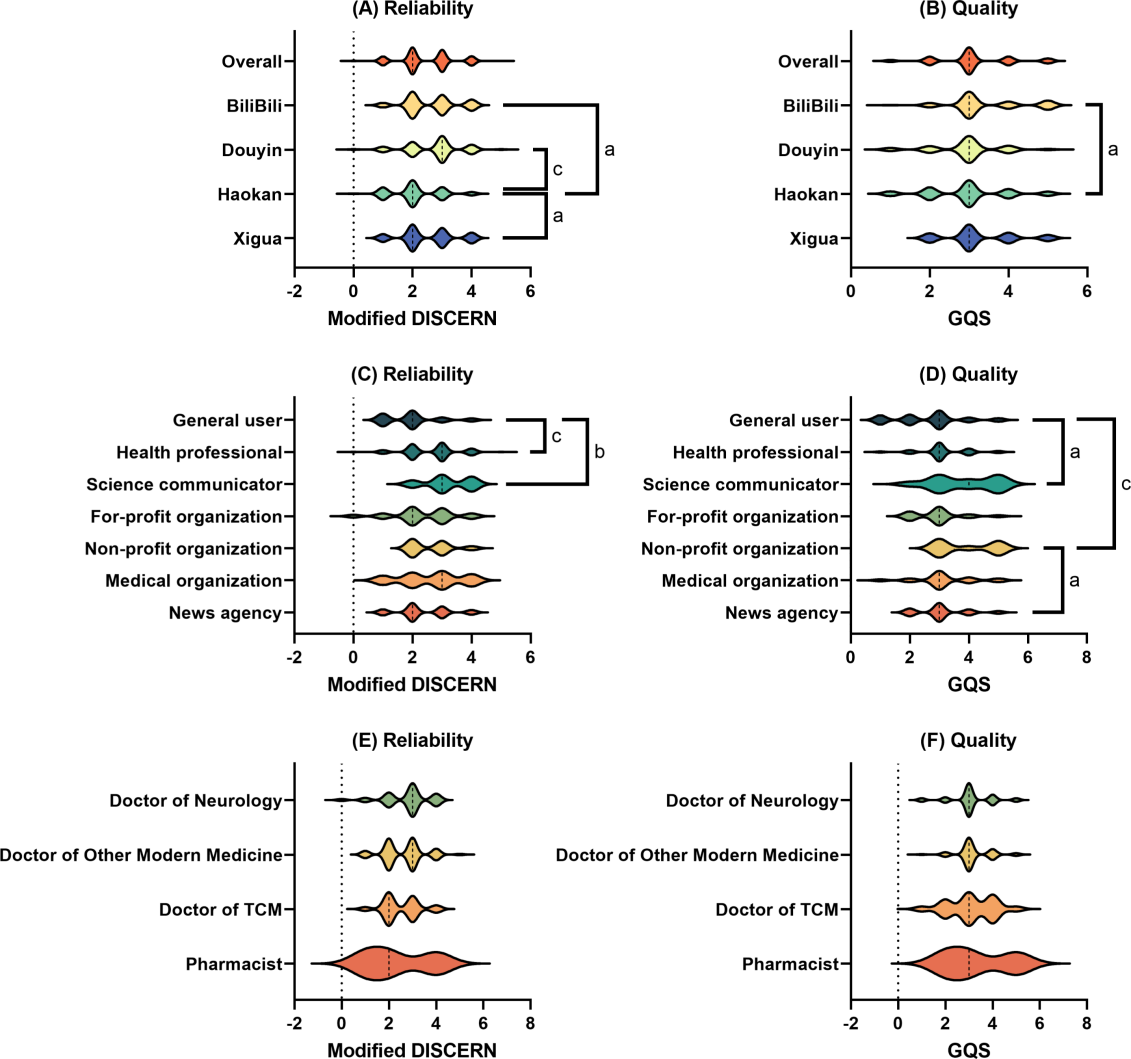
The modified DISCERN and Global Quality Scale (GQS) scores by (A, B) specific video platform, (C, D) video creators, and (E, F) health care professionals. ^a^*P*<.05, ^b^*P*<.01, ^c^*P*<.001. TCM: traditional Chinese medicine.

When examined by specific online video platforms, as shown in Figure 2A and B, our results suggest that videos from Douyin (median modified DISCERN score of 3) exhibited higher median reliability score compared with those from other platforms (median modified DISCERN scores of 2.5, 2, and 2 for Bilibili, Haokan Video, and Xigua Video, respectively). However, this difference was statistically significant only between Douyin and Haokan Video (*P*<.001). Meanwhile, in terms of video quality, the median GQS scores were highly consistent across the 4 video platforms (GQS=3 for all 4 platforms). A statistically significant difference was observed only between Bilibili and Haokan Video (*P*=.03).

### Quality and Reliability by Video Authorship

When analyzed by specific video creator categories, as shown in Figure 2C and D, videos produced by health care professionals (median modified DISCERN score of 3) and science communicators (median modified DISCERN score of 3) demonstrated greater reliability compared with those created by amateur individual users (median modified DISCERN score of 2). However, no statistically significant difference was observed among videos created by different organizational authors (*P*>.99 in all cases). In terms of video quality, the findings were consistent with those for video reliability. Stroke prevention–related videos created by science communicators received higher GQS scores (median GQS score of 4) than those by amateur individual users (median GQS score of 3). No significant difference was observed between videos created by health care professionals and those by amateur users (*P*=.06). Among organizational video creators, videos from nonprofit organizations (median GQS score of 3.5) demonstrated higher quality compared with those from amateur users and news agencies (median GQS score of 3 for both cases).

When the health care professional authorship category was further subdivided (Figure 2E and F), no statistically significant differences were found among videos produced by neurologists (*P*>.99), doctors from other fields of modern medicine (*P*>.99), doctors of TCM (*P*>.99), and pharmacists (*P*>.99), regardless of whether the assessment was based on reliability or quality. However, despite the lack of statistical significance (*P*>.99), videos created by doctors specializing in modern medicine received higher reliability scores than those produced by TCM doctors (median modified DISCERN scores of 3 for both neurologists and other modern medical specialists vs 2 for TCM doctors). Interestingly, pharmacists, though contributing a relatively small proportion of the videos, produced content with moderate quality (median GQS score of 3), possibly reflecting their specialized knowledge in pharmacological prevention strategies, such as the use of antiplatelet agents for stroke risk reduction.

These findings suggest that professional background may influence the rigor of content, even if the differences are not substantial, and underscore the potential value of encouraging more contributions from neurologists and other clinical specialists in stroke-related video creation.

### Video Content Analysis

Regarding the stroke prevention strategies recommended in the included online videos, the vast majority focused on primary prevention (n=262), targeting the first onset of stroke. By contrast, relatively few addressed secondary prevention aimed at preventing stroke recurrence (n=28), and only a small number of videos covered both primary and secondary prevention simultaneously (n=23).

To be more specific, among primary stroke prevention measures, the most frequently mentioned strategy was controlling abnormal blood pressure (158/313, 50.5%), followed by adopting healthy dietary habits (145/313, 46.3%) and engaging in physical activity (144/313, 46.0%). These lifestyle-oriented recommendations reflect prevailing public health messaging trends and align with international stroke prevention guidelines. By contrast, for secondary prevention, the most frequently recommended measure was also managing abnormal blood pressure (24/313, 7.7%), followed by controlling abnormal blood glucose (21/313, 6.7%), regulating abnormal blood lipid levels (21/313, 6.7%), and using oral antiplatelet agents (21/313, 6.7%). This distribution suggests that, where addressed, secondary prevention videos tended to emphasize pharmacological and laboratory-monitored interventions rather than lifestyle-based recommendations.

Collectively, these findings suggest that while primary prevention dominates the landscape of online stroke-related education in China, a considerable gap exists in public-facing content addressing secondary prevention. Enhancing the visibility and clarity of such content, particularly among stroke survivors, may represent a valuable opportunity for public health intervention.

### Correlation of Video General Information With Quality and Reliability

As shown in Table 5, significant correlations were observed among several general video information metrics. In particular, the number of views was positively associated with video length, number of likes, number of shares, and number of comments (*r*=0.142, *P*=.03; *r*=0.838, *P*<.001; *r*=0.897, *P*<.001; and *r*=0.593, *P*<.001, respectively). Similarly, videos that received more likes were more likely to have higher numbers of shares and comments (*r*=0.900, *P*<.001 and *r*=0.816, *P*<.001, respectively).

**Table 5.** The Spearman correlation coefficient between general video information and video quality and reliability.

Correlation	Video Length (s)	Views	Likes	Comments	Shares	Modified DISCERN score	Global Quality Scale score
Video Length (seconds)	1.000	—^a^	—	—	—	—	—
Views (n)	0.142^b^	1.000	—	—	—	—	—
Likes (n)	−0.060	0.838^b^	1.000	—	—	—	—
Comments (n)	−0.045	0.593^b^	0.816^b^	1.000	—	—	—
Shares (n)	−0.017	0.897^b^	0.900^b^	0.826^b^	1.000	—	—
Modified DISCERN scores	0.151^b^	−0.105	0.027	0.137^b^	0.019	1.000	—
Global Quality Scale score	0.441^b^	−0.042	−0.077	−0.017	−0.110	0.559^b^	1.000

a Not applicable.

b Statistical significance (*P*<.05).

Nevertheless, when considering video quality and reliability, video length was found to be positively correlated with both the modified DISCERN and GQS scores (*r*=0.151, *P*=.007 and *r*=0.441, *P*<.001, respectively). Additionally, a greater number of comments was associated with higher video reliability, although this correlation was relatively weak (*r*=0.137, *P*=.01).

### Temporal Trend in Video Quality and Reliability

According to Figure S1 in Multimedia Appendix 1, an analysis of video upload time revealed a slight but statistically significant positive correlation between more recent upload dates and both video quality (*P*=.002) and reliability (*P*=.01). Specifically, Pearson correlation coefficients indicated that videos uploaded more recently tended to have higher GQS scores (*r*=0.113, *P*=.002) and higher modified DISCERN scores (*r*=0.086, *P*=.01). Although the strength of these associations was relatively weak, the findings suggest a gradual improvement in the overall quality and reliability of stroke-related video content over time. This trend may reflect growing awareness among content creators regarding evidence-based health communication, as well as improved platform policies aimed at enhancing the accuracy of health information.

## Discussion

### Principal Findings

In this study, by searching and screening online videos related to stroke prevention across 4 popular Chinese-language video platforms, we systematically described and analyzed the general characteristics, as well as the overall quality and reliability, of the included health-related online videos. To the best of our knowledge, this may be one of the first studies to specifically evaluate and report on the quality and reliability of Chinese-language online video platforms as information sources for stroke prevention education in Mainland China. We hope that our findings will provide valuable data for real-world policy makers and medical practitioners, ultimately contributing to improved stroke prevention and management efforts in the future.

Overall, using the modified DISCERN and GQS instruments, the included online videos related to stroke prevention were evaluated as relatively unreliable and of moderate quality. This suggests that, although video creators may offer some useful advice on primary and secondary stroke prevention measures, they often fail to provide supporting references and frequently lack fairness and objectivity in presenting information. These findings are consistent with previous research in other medical domains, which has similarly shown that health-related content on new media, particularly online video-sharing platforms, is often unsatisfactory and, at times, misleading [40-43]. This phenomenon may be largely attributed to the competition for audience attention in the digital marketplace, where accurate scientific information, often complex and less engaging for the general public, is easily overshadowed by sensationalized or simplified claims [44]. Consequently, entertainment-oriented online platforms are more likely to recommend light, engaging content over scientifically rigorous materials, which may be more difficult for general audiences to understand. Nevertheless, a growing number of stroke-related educational videos are being created and uploaded each year, suggesting that stroke prevention is becoming an increasingly visible topic on Chinese social media, particularly on video-sharing platforms. Notably, a positive temporal trend was observed in the quality and reliability of stroke-related videos uploaded between 2017 and 2023. We speculate that this improvement may be driven by increased participation from health care professionals in content creation, thereby directly contributing to the enhanced quality of stroke prevention information available online.

When analyzed by specific video platforms, videos from Douyin appeared to be more favorable, exhibiting higher content quality and reliability compared with those from the other 3 platforms, although statistical significance was not consistently observed. This finding may be explained by the fact that approximately 81% of eligible videos on Douyin were created and uploaded by qualified health care professionals, who generally receive more comprehensive and systematic evidence-based medical education and training than general users. Therefore, this phenomenon is relatively understandable. However, when the data for health care professionals were further stratified into more specific subgroups, videos created by doctors specializing in modern medicine—regardless of their field—tended to be more reliable than those produced by doctors of TCM, despite the absence of statistically significant differences.

Unfortunately, alongside the potentially beneficial health-related recommendations found in stroke prevention videos, concerns have also been raised about the prevalence of misinformation in the included study videos. Notably, some general individual users without a medical education background may share personal opinions in their videos that lack solid scientific evidence. For example, one video created by amateurs claimed that “regularly receiving intravenous injections can prevent stroke onset because it may help clear blood vessels.” This statement has been identified as a widely circulated misconception among the Chinese population, particularly among older adults. Additionally, some video creators were observed to use emotionally charged and sensationalized titles and content, rather than objective and evidence-based information, in an effort to attract public attention and maximize viewership. However, this approach may lead to severe misunderstandings that could potentially jeopardize public health at the community level. Furthermore, the risk of misinformation is not limited to nonprofessionals, as certain content produced by health care professionals also warrants scrutiny. Specifically, current stroke prevention recommendations and suggestions from TCM doctors are highly heterogeneous, with some approaches not yet completely validated by formal clinical trials and largely based on empirical knowledge. For instance, some TCM practitioners propose that massage on specific body points (commonly referred to as “acupoints”) may help prevent cerebral stroke. While this perspective is rooted in traditional practice, there is currently insufficient evidence from randomized controlled trials to substantiate its efficacy. Nevertheless, despite TCM’s historically experience-driven approach, it remains an integral component of China’s health care system and continues to serve as a complementary and alternative option in stroke prevention and management. In recent decades, TCM practitioners have made concerted efforts to integrate traditional practices with modern scientific research, contributing to its ongoing development and potential role in future stroke care [45].

To effectively address these issues, several targeted countermeasures can be implemented to enhance the quality and reliability of health-related online videos and information [46-48]. First and foremost, the review process for medical information videos uploaded to online platforms should be reinforced and supervised in collaboration with qualified medical professionals. Additionally, establishing and strengthening an online monitoring and correction system could help limit the spread of misinformation and reduce its long-term impact. Finally, general social media users should be encouraged to share controversial health information more selectively and cautiously, in order to curb the dissemination of false content and restrict accounts that produce or promote it.

Recently, Chinese governmental departments and relevant enterprises have begun taking active measures to combat the widespread dissemination of health-related misinformation online. For example, in collaboration with the Ministry of Industry and Information Technology, the Chinese National Health Commission issued a policy on May 27, 2024, aimed at urgently strengthening the regulation of health-related webcasting, video content, and promotional activities on digital platforms. Simultaneously, ByteDance (TikTok’s parent company) launched a centralized governance initiative in 2024 focused on identifying and removing poor-quality or even fraudulent sources of medical information. Beyond China, the World Health Organization (WHO) has proposed several countermeasures to limit online misinformation [49]. These include awareness campaigns targeting patients and health care professionals, the promotion of platforms that provide evidence-based data, the integration of scientific evidence into health-related content in mass media, and efforts to improve media and health literacy. Additionally, the WHO encouraged the active involvement of experts and health care professionals in debunking misinformation and guiding internet users toward credible, evidence-based information sources. However, the persistence of misinformation in the digital space, particularly in health care, continues to pose a significant threat to public well-being worldwide. This ongoing challenge underscores the urgent need for stronger guidelines and innovative strategies to mitigate its impact effectively.

In addition to evaluating the quality and characteristics of stroke-related videos, it is important to consider the platform-specific policies that may influence the dissemination of health content. Major Chinese video-sharing platforms, such as Douyin, have publicly issued guidelines encouraging the distribution of accurate and authoritative health information. For instance, these platforms typically require health-related content creators to verify their professional credentials and may implement content moderation mechanisms to flag or remove misleading medical information. However, the enforcement and transparency of these policies vary across platforms, and the extent to which they affect the quality and reach of health-related videos remains largely understudied. Future research could benefit from a more systematic analysis of how platform-level governance affects the credibility and visibility of online health communication.

### Limitations

Despite these findings, several limitations of this study should be acknowledged. First and most importantly, our research team retrieved and analyzed only Chinese-language videos targeting Chinese audiences, using a limited set of search keywords. Videos in other languages, such as English, Japanese, and Korean, were not included, indicating a need for broader investigation in future research. Second, although measures were taken to minimize potential bias introduced by platform algorithms during the initial video search, we cannot fully exclude the possibility that our results were influenced by the artificial intelligence algorithms used by commercial video platforms. To be more specific, video search engines tend to prioritize and recommend content with higher engagement metrics. Although we logged out of our accounts, deleted temporary files and cookies, and conducted searches using a newly installed internet browser, the inherent nature of these automatic algorithms amplifies disparities between videos—allowing popular content to gain increasing visibility, while less popular videos receive minimal exposure. Consequently, this unavoidable limitation may have influenced the collection of general video-related metrics, particularly likes and shares. Third, this study was based on a limited sample size (from only 4 Chinese domestic video platforms). Therefore, further research involving a larger number of videos and platforms with broader representativeness is urgently needed. Fourth, data collection was conducted on a single day, which may not fully capture the dynamic and evolving nature of content rankings on video platforms. Videos that consistently maintain high visibility over time might have been overlooked, and our findings may therefore represent only a snapshot of what users were most likely to encounter at that specific moment. Future studies could incorporate longitudinal sampling strategies to account for temporal variations in content prominence. Finally, as a cross-sectional study using a relatively subjective evaluation strategy, this study primarily focused on a descriptive assessment of the current state of online video-based patient education in Mainland China. Therefore, future prospective studies are warranted to evaluate the real-world effectiveness of physician-created and peer-reviewed video materials for patient education.

### Conclusion

This study examined the overall quality and reliability of Chinese-language videos related to public patient education on stroke prevention. The findings suggest that the quality and reliability of health-related videos, as a source of information for stroke management, remain relatively unsatisfactory and should be approached with caution by Chinese audiences. Therefore, targeted countermeasures, such as reinforcing the video content review process, establishing online correction mechanisms, and encouraging users to selectively and carefully share controversial information, are urgently needed to enhance the credibility and impact of stroke-related educational content in the future.

## Supplementary material

10.2196/64891 Multimedia Appendix 1 Description of author identity of included online videos and the temporal trends of modified DISCERN and Global Quality Scale scores of Chinese language stroke prevention–related videos.

## Abbreviations

CCTV: China Central Television
GQS: Global Quality Scale
TCM: traditional Chinese medicine
WHO: World Health Organization
