# Supplementary material for: The Quality and Reliability of Online Videos as an Information Source of Public Health Education for Stroke Prevention in Mainland China: Electronic Media–Based Cross-Sectional Study
Source: JMIR Infodemiology. 2025 Jul 21;5:e64891. doi: 10.2196/64891 (PMC12303359; doi:10.2196/64891)
Supplement: Multimedia Appendix 1 [file infodemiology-v5-e64891-s001.docx]

| **Supplementary Table 1.** The description of author identity of included online videos | |
| --- | --- |
| **Author Identity** | **Definition and Description** |
| **General Classification** | |
| General Users | Individuals who do not have real name recognition or professional accreditation in any medical area. |
| Health Professionals | Individuals who have real name recognition and professional accreditation in certain medical area, including doctors, nurses and other health-related professionals. |
| Science Communicators | Individuals who are able to utilize their high level of scientific literacy to communicate concepts in formats which are accessible, accurate, and appealing to a desired demographic. |
| News Agencies | Including public or private newspaper, TV station, and network media without professional accreditation in medical area. |
| For-profit Organizations | For-profit organizations with fully or partly commercial backgrounds, including medical companies that provide online consulting and health-related information communication service and any other commercial groups with for-profit purposes. |
| Non-profit Organizations | Non-profit organizations with no commercial backgrounds, including patient community, science popularization project with public welfare purposes, and any other non-profit groups. |
| Medical Organizations | Healthcare institutions established in accordance with local legal procedures to engage in disease diagnosis and treatment activities. |
| **Further Classification for Health Professionals** | |
| Doctors Specializing in Neurology of Modern Evidence-Based Medicine | Doctors licensed to specialize in neurology, particularly stroke or cerebrovascular disease, of modern evidence-based medicine. |
| Doctors Specializing in Other Areas of Modern Evidence-Based Medicine | Doctors licensed to specialize in other internal medicine, surgery, dental medicine, critical care medicine, emergency medicine, imaging, interventional medicine, rehabilitation medicine, and any other areas of modern evidence-based medicine. |
| Doctors of Traditional Chinese Medicine (TCM) | Doctors licensed to specialize in Chinese traditional medicine which is related with herbal medicine, massage, acupuncture, moxibustion, and other traditional treatment measures. |
| Pharmacists | Healthcare professionals licensed to engage in pharmacy with duties including dispensing prescription drugs, monitoring drug interactions, administering vaccines, and counseling patients regarding the effects and proper usage of drugs and dietary supplements. |

**
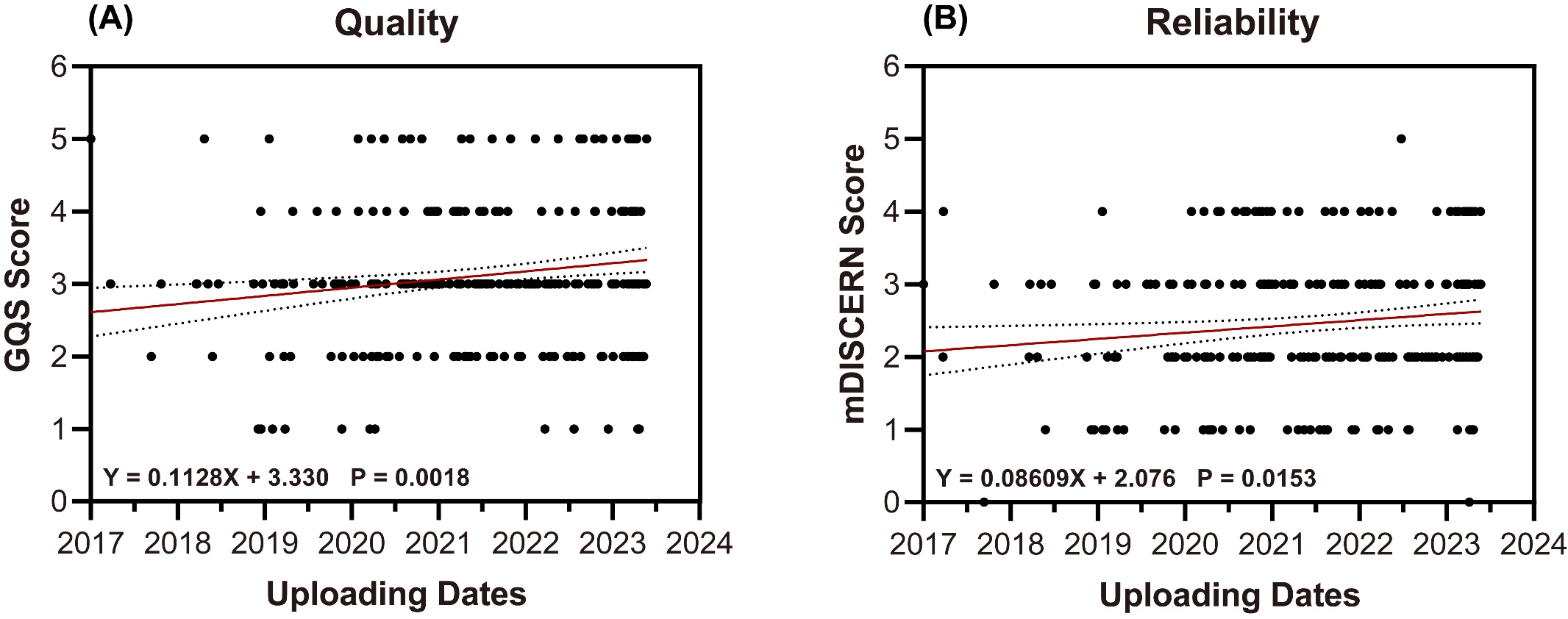
Supplementary Figure 1.** The temporal trends of modified DISCERN and GQS scores of Chinese language stroke prevention-related video from 2017 to 2023
